# Determinants of first-line clinical trial enrollment among Black and White gynecologic cancer patients

**DOI:** 10.1007/s10552-025-01963-y

**Published:** 2025-02-03

**Authors:** Autumn B. Carey, Caitlin E. Meade, Britton Trabert, Casey M. Cosgrove, Ashley S. Felix

**Affiliations:** 1 Division of Epidemiology, College of Public Health, The Ohio State University, Columbus, OH USA; 2 Department of Obstetrics and Gynecology, Spencer Fox Eccles School of Medicine, University of Utah, Salt Lake City, UT USA; 3 Huntsman Cancer Institute, University of Utah, Salt Lake City, UT USA; 4 Department of Obstetrics and Gynecology, Division of Gynecologic Oncology, Arthur G James Cancer Center, The Ohio State University Wexner Medical Center, Columbus, OH USA

**Keywords:** Hospital-based cancer registry, Health disparities, Clinical trials, Minority enrollment

## Abstract

**Purpose:**

Disparities in gynecologic cancer clinical trial enrollment exist between Black and White patients; however, few examine racial differences in clinical trial enrollment predictors. We examined whether first-line clinical trial enrollment determinants differed between Black and White gynecologic cancer patients.

**Methods:**

We used the National Cancer Database to identify Black and White gynecologic cancer (cervix, ovarian, uterine) patients diagnosed in 2014–2020. Multivariable logistic regression was used to estimate adjusted odds ratios (ORs) and 95% confidence intervals (CIs) for associations between clinical trial enrollment (yes vs no) and sociodemographic, facility, tumor, and treatment characteristics stratified by race. We included a multiplicative interaction term between each assessed predictor and race to test whether associations differed by race.

**Results:**

We included 703,022 gynecologic cancer patients (mean [SD] age at diagnosis, 60.9 [13.1] years). Clinical trial enrollment was lower among Black (49/86,058, 0.06%) vs. White patients (710/616,964, 0.11%). Only cancer site differed by race: among Black patients, a cervical vs. uterine cancer diagnosis (OR = 4.63, 95% CI = 1.67–12.88) was associated with higher clinical trial enrollment odds, while among White patients, both cervical (OR = 2.21, 95% CI = 1.48–3.29) and ovarian (OR = 3.40, 95% CI = 2.58–4.47) cancer diagnoses (vs. uterine cancer) were associated with higher enrollment odds. Most predictors were associated with clinical trial enrollment odds among White but not Black patients.

**Conclusion:**

Few differences in first-line clinical trial enrollment predictors exist between Black and White gynecologic cancer patients. Although small numbers of Black patients and low clinical trial prevalence are limitations, this descriptive analysis is important in understanding racially disparate clinical trial enrollment.

**Supplementary Information:**

The online version contains supplementary material available at 10.1007/s10552-025-01963-y.

## Introduction

Cancer-related clinical trials are essential for ensuring validity, generalizability, and equity of cancer treatment as well as advancing medical knowledge. For adults with cancer, a systematic review of National Clinical Trials Network (NCTN) clinical trials revealed significant gains in population life-years, a documented influence on cancer treatment guidelines, and low federal cost per life-year gained, signaling the population and clinical impacts of clinical trials [1]. Despite evidence that cancer patients are willing to participate in clinical trials [2], less than 7% of US adults with cancer participate in clinical trials [3]. This mismatch between intention and reality is likely the result of complex interactions between barriers operating at the structural, clinical, provider, and patient levels [4]. Compounding the overall low prevalence of clinical trial enrollment among adults with cancer are the known racial and ethnic disparities showing lower participation among historically underrepresented groups [5, 6]. Understanding the specific barriers that disproportionately limit participation of minoritized cancer patients in clinical trials is a necessary step to identifying leverage points for future interventions.

Within the gynecologic cancer population, enduring disparities in clinical trial participation are evident [7–14]. Although racially minoritized compared with White gynecologic cancer patients are less likely to participate in clinical trials, few studies characterize race-specific determinants that underlie these disparities. Limited English language proficiency has been identified as a predictor of lower clinical trial enrollment odds among gynecologic cancer patients, with some evidence of effect modification by race and ethnicity [15]. Building on this work, we explored whether associations between demographic, area-level, tumor, and treatment characteristics and clinical trial enrollment during first line of treatment differed between Black and White gynecologic cancer patients.

## Methods

### Data source

The 2021 Participant User File (PUF) was obtained from the hospital-based National Cancer Database (NCDB), [16] a cancer registry capturing 70% of cancers diagnosed in the United States. The registry data includes sociodemographic characteristics, tumor characteristics, treatment facility attributes, treatment, and survival outcomes abstracted from patient medical records by Certified Tumor Registrars [17]. Data submitted to NCDB undergo rigorous quality checks according to American College of Surgeons standards. This study was exempt from the OSU Institutional Review Board, and we followed the Strengthening the Reporting of Observational Studies in Epidemiology (STROBE) reporting guideline [18].

### Study population

We conducted a retrospective cohort study of patients diagnosed with one of the following primary gynecologic cancers [International Classification of Disease for Oncology, Third Edition (ICD-O-3) primary site codes: uterine (C54.0-C54.9, C55.9), cervical (C53.0, C53.1, C53.8, C53.9), ovarian (C56.9), fallopian tube (C57.0), and peritoneal (C48.1, C48.2)] between 2004 and 2020 using data from the NCDB. We identified 872,621 patients (female sex, ≥ 18 years of age) who self-reported non-Hispanic White (hereafter, White) or non-Hispanic Black (hereafter, Black) race. From this overall study population, we excluded patients with unknown clinical trial enrollment status (*n* = 71), non-invasive stage (*n* = 163,961), and cancer site-specific surgery procedures (*n* = 5,567, Online Resource 1). After exclusions, 703,022 patients remained in the analytic sample. Ovarian, peritoneal, and fallopian tube cancers were grouped together as ovarian cancer for analysis.

### First-line clinical trial enrollment

We used the NCDB variable RX_SUMM_OTHER and categorized patients as enrolled in a clinical trial during first line of treatment when the response observations were enrolled in an institutional (code 2) or double-blind clinical trial (code 3). Categories of no trial (code 0), other (code 1), other – unproven (code 6), or refused trial (code 7) were categorized as not enrolled in a clinical trial [19]. These categories represented 99.78%, 0.20%, 0.02%, and < 0.01%, respectively, of the total “not enrolled” category (*n* = 702,263).

### Covariates

Covariates included: race (Black, White), age at diagnosis (continuous), Charlson-Deyo comorbidity score (0, 1, or ≥ 2), health insurance (none, private, Medicaid, Medicare, other government, unknown), distance to treatment facility (in miles; tertiles based on the analytic study population < 7.6, 7.6–23.4, > 23.4, unknown), area-level income quartiles (< $46,277, $46,277-$57,856, $57,857-$74,062, ≥ 74,063, unknown), area-level educational attainment quartiles (measure of the number of adults who did not graduate from high school; ≥ 15.3%, 9.1–15.2%, 5–9%, < 5%, unknown), metro status [urban, large metro county (population > 1 million), medium to small metro county (population < 1 million), rural, unknown], facility location (Midwest, Mountain, Northeast, Pacific, South, unknown), facility type (community cancer, comprehensive community cancer, academic/research, integrated network cancer, unknown), year of diagnosis (2004–2007, 2008–2011, 2012–2015, 2016–2020), surgery (yes, no), chemotherapy (yes, no, unknown), radiation (yes, no, unknown), grade (1, 2, 3, applicable for uterine and ovarian endometrioid and ovarian serous only), stage (I, II, III, IV), and cancer site (cervix, ovary, uterine). For most variables, the largest category was modeled as the reference group, *e.g.,* uterine cancer was the reference group for the cancer site variable. For other variables, we used the most intuitive category as the reference group, *e.g.,* shortest distance from the treating facility was the reference for the distance from facility variable.

Area-level income and education for each patient’s area of residence is estimated by matching the patient’s zip code with 2020 American Community Survey data, spanning years 2016–2020 and adjusted for 2020 inflation. We categorized stage (I, II, III, IV) using the American Joint Commission on Cancer pathological and/or clinical staging criteria. We prioritized pathologic staging criteria for all cancer sites based on clinical practice. For cervical or ovarian cancer patients with missing pathologic stage, we supplemented with clinical stage.

### Statistical analysis

We used multivariable logistic regression to estimate adjusted odds ratios (ORs) and 95% confidence intervals (CIs) for associations between clinical trial enrollment during first line of treatment and sociodemographic, facility-based characteristics in the overall study population and stratified by race (*i.e.,* Black and White patients separately). We included a multiplicative interaction term between each assessed predictor and race in an overall multivariable model to test whether associations differed by race.

All variables shown in Table 1 were associated with clinical trial enrollment in univariable models (*p* < 0.05, data not tabled) and were included in the multivariable models. In the main models, variables with missing data were included in the models as a separate missing category. Sensitivity analyses were conducted excluding individuals with missing covariate data (34.6% of the main sample was excluded) and utilizing multiple imputation by chained equations (MICE), also known as fully conditional specification and sequential regression multivariate imputation, to impute the missing and unknown data. MICE generates imputations based on a set of imputation models for each variable with missing values and imputes different variable types, such as continuous, binary, and categorical data. The MICE model was specified to complete 5 imputations with 5 iterations [20–22]. Effect estimates were computed on each imputed dataset and pooled using Rubin’s rules [20, 23, 24]. Statistical analyses were performed using SAS (version 9.4, SAS Institute, Cary, NC, USA) or R (version 4.4.1). All *P* values were two-sided; statistical significance was set at *P* less than 0.05.

**Table 1:** Multivariable-adjusted odds ratios (ORs) and 95% confidence intervals (CIs) for associations between epidemiologic, facility, and tumor, and first-line treatment characteristics and clinical trial enrollment by race

	Black (*n* = 86,058)				White (*n* = 616,964)				
	Not Enrolled (*n* = 86,009)	Enrolled (*n* = 49)	OR (95% CI)^a^	p	Not Enrolled (*n* = 616,254)	Enrolled (*n* = 710)	OR (95% CI)^a^	p	P_int_^b^
Age, mean (SD)	59.44 (13.39)	56.98 (12.50)	1.02 (0.85, 1.21)	0.87	61.11 (13.09)	59.86 (11.06)	0.91 (0.86, 0.95)	< 0.0001	0.72
Charlson-Deyo score				0.34				0.01	0.15
0	62,077 (72.2)	33 (67.4)	1.00		480,188 (77.9)	599 (84.4)	1.00		
1	17,050 (19.8)	12 (24.5)	1.64 (0.84, 3.22)		102,531 (16.6)	94 (13.2)	0.94 (0.76, 1.18)		
≥ 2	6,882 (8.0)	4 (8.2)	1.35 (0.47, 3.88)		33,535 (5.4)	17 (2.4)	0.49 (0.30, 0.79)		
Insurance Status				0.14				0.19	0.49
No insurance	5,400 (6.3)	4 (8.2)	0.88 (0.30, 2.59)		18,239 (3.0)	16 (2.3)	0.84 (0.50, 1.39)		
Private insurance	33,459 (38.9)	26 (53.1)	1.00		303,199 (49.2)	404 (56.9)	1.00		
Medicaid	13,525 (15.7)	10 (20.4)	0.65 (0.31, 1.39)		39,552 (6.4)	38 (5.4)	0.71 (0.51, 1.00)		
Medicare	30,886 (35.9)	8 (16.3)	0.28 (0.11, 0.69)		239,950 (38.9)	240 (33.8)	0.87 (0.70, 1.08)		
Other government	903 (1.1)	1 (2.0)	1.24 (0.16, 9.41)		6,289 (1.0)	7 (1.0)	0.87 (0.41, 1.85)		
Distance from facility, miles				0.12				0.0002	0.11
< 7.6 miles	35,490 (41.3)	23 (46.9)	1.00		168,661 (27.4)	139 (19.6)	1.00		
7.6–23.4 miles	23,869 (27.8)	8 (16.3)	0.58 (0.25, 1.35)		184,882 (30.0)	235 (33.1)	1.35 (1.09, 1.67)		
> 23.4 miles	16,723 (19.4)	13 (26.5)	1.94 (0.81, 4.66)		195,042 (31.7)	261 (36.8)	1.61 (1.29, 2.02)		
Area-level income				0.14				0.43	0.15
Quartile 1: < $46,277	31,394 (36.5)	20 (40.8)	1.00		73,582 (11.9)	59 (8.3)	1.00		
Quartile 2: $46,277-$57,856	16,738 (19.5)	14 (28.6)	0.97 (0.45, 2.09)		120,750 (19.6)	121 (17.0)	1.06 (0.77, 1.47)		
Quartile 3: $57,857-$74,062	13,052 (15.2)	5 (10.2)	0.37 (0.12, 1.10)		135,939 (22.1)	138 (19.4)	0.87 (0.62, 1.22)		
Quartile 4: ≥ $74,063	13,853 (16.1)	5 (10.2)	0.27 (0.08, 0.95)		211,110 (34.3)	306 (43.1)	0.85 (0.59, 1.22)		
Area-level education (% without high school diploma)				0.06				0.01	0.49
Quartile 1: < 5%	5,096 (5.9)	2 (4.1)	1.00		131,414 (21.3)	209 (29.4)	1.00		
Quartile 2: 5%—9%	13,108 (15.2)	13 (26.5)	1.77 (0.37, 8.38)		172,338 (28.0)	209 (29.4)	0.84 (0.68, 1.03)		
Quartile 3: 9.1%−15.2%	26,787 (31.1)	15 (30.6)	0.64 (0.13, 3.26)		151,879 (24.7)	135 (19.0)	0.64 (0.50, 0.83)		
Quartile 4: ≥ 15.3%	30,203 (35.1)	14 (28.6)	0.45 (0.08, 2.47)		87,459 (14.2)	74 (10.4)	0.64 (0.46, 0.88)		
Metro status				0.48				0.0001	0.71
Large metro county (population > 1 million)	56,975 (66.2)	36 (73.5)	1.00		292,952 (47.5)	398 (56.1)	1.00		
Medium/small metro county (population < 1 million)	19,093 (22.2)	7 (14.3)	0.46 (0.19, 1.13)		193,354 (31.4)	197 (27.8)	0.76 (0.63, 0.91)		
Urban	7,001 (8.1)	4 (8.2)	0.69 (0.20, 2.43)		92,784 (15.1)	72 (10.1)	0.55 (0.41, 0.73)		
Rural	896 (1.0)	1 (2.0)	1.35 (0.16, 11.50)		11,323 (1.8)	13 (1.8)	0.88 (0.49, 1.56)		
Facility location				0.73				0.004	0.56
Northeast	15,347 (17.8)	11 (22.5)	1.00		127,986 (20.8)	211 (29.7)	1.00		
South	44,393 (51.6)	20 (40.8)	0.61 (0.26, 1.39)		193,695 (31.4)	185 (26.1)	0.68 (0.55, 0.84)		
Midwest	14,028 (16.3)	13 (26.5)	1.08 (0.46, 2.54)		160,870 (26.1)	169 (23.8)	0.73 (0.59, 0.90)		
Mountain	639 (0.7)	0 (0.0)	NE		27,759 (4.5)	36 (5.1)	0.96 (0.66, 1.38)		
Pacific	3,909 (4.5)	1 (2.0)	0.50 (0.06, 4.04)		65,926 (10.7)	73 (10.3)	0.78 (0.59, 1.03)		
Facility type				0.17				< 0.0001	0.77
Community Cancer Program	2,097 (2.4)	1 (2.0)	1.00		21,740 (3.5)	12 (1.7)	1.00		
Comprehensive Community Cancer Program	21,768 (25.3)	5 (10.2)	0.46 (0.05, 4.02)		211,910 (34.4)	144 (20.3)	1.06 (0.59, 1.92)		
Academic/Research Program	38,164 (44.4)	31 (63.3)	1.30 (0.18, 9.73)		220,842 (35.8)	424 (59.7)	2.48 (1.38, 4.43)		
Integrated Network Cancer Program	16,287 (18.9)	8 (16.3)	0.83 (0.10, 6.78)		121,744 (19.8)	94 (13.2)	1.11 (0.61, 2.04)		
Diagnosis year				0.04				< 0.0001	0.10
2004–2007	13,073 (15.2)	1 (2.0)	1.00		109,808 (17.8)	44 (6.2)	1.00		
2008–2011	17,791 (20.7)	11 (22.5)	8.09 (1.04, 62.77)		133,798 (21.7)	103 (14.5)	1.98 (1.39, 2.81)		
2012–2015	23,009 (26.8)	11 (22.5)	6.13 (0.79, 47.77)		162,534 (26.4)	117 (16.5)	1.89 (1.33, 2.67)		
2015–2020	32,136 (37.4)	26 (53.1)	11.63 (1.56, 86.52)		210,114 (34.1)	446 (62.8)	6.11 (4.47, 8.36)		
Cancer site				0.01				< 0.0001	0.003
Uterine	45,526 (52.9)	12 (24.5)	1.00		345,223 (56.0)	94 (13.2)	1.00		
Ovarian	19,662 (22.9)	21 (42.9)	1.73 (0.78, 3.82)		184,190 (29.9)	564 (79.4)	3.40 (2.58, 4.47)		
Cervical	20,821 (24.2)	16 (32.7)	4.63 (1.67, 12.88)		86,841 (14.1)	52 (7.3)	2.21 (1.48, 3.29)		
Tumor stage				0.001				< 0.0001	0.85
I	40,923 (47.6)	4 (8.2)	1.00		343,153 (55.7)	43 (6.1)	1.00		
II	9,012 (10.5)	3 (6.1)	2.51 (0.51, 12.32)		50,754 (8.2)	49 (6.9)	4.71 (3.03, 7.31)		
III	21,043 (24.5)	26 (53.1)	8.84 (2.68, 29.12)		144,212 (23.4)	405 (57.0)	9.88 (6.88, 14.19)		
IV	15,031 (17.5)	16 (32.7)	7.44 (2.15, 25.80)		78,135 (12.7)	213 (30.0)	10.28 (7.06, 14.97)		
Surgery				0.09				< 0.0001	0.08
No surgery	17,544 (20.4)	13 (26.5)	1.00		71,406 (11.6)	61 (8.6)	1.00		
Any surgery	68,465 (79.6)	36 (73.5)	2.09 (0.90, 4.85)		544,848 (88.4)	649 (91.4)	2.57 (1.90, 3.47)		
Chemotherapy				0.09				< 0.0001	1.00
No	42,889 (49.9)	6 (12.2)	1.00		352,448 (57.2)	68 (9.6)	1.00		
Yes	41,267 (48.0)	43 (87.8)	2.98 (1.11, 8.00)		253,025 (41.1)	642 (90.4)	2.15 (1.60, 2.87)		
Radiation				0.32				0.05	0.12
No	55,286 (64.3)	34 (69.4)	1.00		446,174 (72.4)	616 (86.8)	1.00		
Yes	27,886 (32.4)	15 (30.6)	0.51 (0.21, 1.24)		150,928 (24.5)	76 (10.7)	0.69 (0.49, 0.97)		

^a^Unknown categories are included in the model but omitted from the table

^b^interaction between race and the assessed variable

*NE* Not estimable

Odds ratios greater than 1.00 indicate higher odds of clinical trial enrollment associated with the factor while odds ratios less than 1.00 indicate lower odds of clinical trial enrollment associated with the factor compared with the reference category

## Results

Among the 703,022 gynecologic cancer patients included (mean [standard deviation] age at diagnosis, 60.9 [13.1] years), 759 (0.11%) were enrolled in a clinical trial during first line of treatment. Table 1 shows the distribution of epidemiological, facility, tumor, and treatment characteristics by clinical trial enrollment and multivariable-adjusted associations with clinical trial enrollment stratified by race. Lower first-line clinical trial enrollment among Black (*n* = 49/86,058, 0.06%) vs. White (*n* = 710/616,964, 0.11%) patients was noted. Most predictors did not differ according to race. Only cancer site was differently associated with first-line clinical trial enrollment odds (*p interaction* = 0.003): among Black patients, a cervical vs. uterine cancer diagnosis (OR = 4.63, 95% CI = 1.67–12.88) was associated with higher first-line clinical trial enrollment odds, while among White patients, both cervical (OR = 2.21, 95% CI = 1.48–3.29) and ovarian (OR = 3.40, 95% CI = 2.58–4.47) cancer diagnoses were associated with higher enrollment odds vs. a uterine cancer diagnosis. Other characteristics associated with first-line clinical trial enrollment among Black patients included more recent year of diagnosis (OR for diagnosis in 2015–2020 vs. 2004–2007 = 11.63, 95% CI = 1.56–86.52) and advanced stage (III vs. I OR = 8.84, 95% CI = 2.68–29.12; IV vs. I OR = 7.44, 95% CI = 2.15–25.80). We noted certain additional characteristics were related to clinical trial enrollment among Black patients: Having Medicare vs. private insurance (OR = 0.28, 95% CI = 0.11–0.69), living in zip codes with higher vs. lower area-level income (Q4 vs. Q1 OR = 0.27, 95% CI = 0.08–0.95) were inversely associated with first-line clinical trial enrollment, while concurrent chemotherapy was associated with higher enrollment odds (OR = 2.98, 95% CI = 1.11–8.00).

Among White gynecologic cancer patients, older age at diagnosis (OR per 5-year increment = 0.91, 95% CI = 0.86–0.95) and having 2 or more vs. no comorbidities (OR = 0.49, 95% CI = 0.30–0.79) were associated with lower odds of first-line clinical trial enrollment. Area-level characteristics including living in zip code areas with lower area-level educational attainment (Q4 vs. Q1 OR = 0.64, 95% CI = 0.46–0.88) and medium/small (OR = 0.76, 95% CI = 0.63–0.91) or urban (OR = 0.55, 95% CI = 0.41–0.73) counties vs. large metros were associated with lower odds of clinical trial enrollment during first line of treatment among White gynecologic cancer patients. Living further from the treatment facility (7.6–23.4 vs. < 7.6 miles: OR = 1.35, 95% CI = 1.09–1.67; > 23.4 vs. < 7.6 miles: OR = 1.61, 95% CI = 1.29–2.02) was associated with higher odds of clinical trial enrollment among White gynecologic cancer patients. Facility-level characteristics associated with clinical trial enrollment among White gynecologic cancer patients included facility type and location. Treatment at an academic or research program vs. a community cancer program (OR = 2.48, 95% CI = 1.38–4.43) was associated with higher clinical trial enrollment while treatment in the South (OR = 0.68, 95% CI = 0.55–0.84) or Midwest (OR = 0.73, 95% CI = 0.59–0.90) compared with the Northeast was associated with lower odds of clinical trial enrollment among White gynecologic cancer patients.

Clinical trial enrollment during first line of treatment among White gynecologic cancer patients increased over the study period (diagnosis in 2015–2020 vs. 2004–2007 OR = 6.11, 95% CI = 4.47–8.36). Advanced stage (III vs. I OR = 9.88, 95% CI = 6.88–14.19; IV vs. I OR = 10.28, 95% CI = 7.06–14.97), receipt of surgery (OR = 2.57, 95% CI = 1.90–3.47), and receipt of chemotherapy (OR = 2.15, 95% CI = 1.60–2.87) were associated with higher odds of clinical trial enrollment among White gynecologic cancer patients, while radiation was associated with lower clinical trial enrollment odds (OR = 0.69, 95% CI = 0.49–0.97).

We ran sensitivity analyses excluding individuals with missing covariate data and utilizing MICE to evaluate the impact of missing data on our results. In models where participants with missing data were excluded (Online Resource 2), we observed additional associations among Black patients, namely distance from facility, area-level income, area-level education, metro status, and chemotherapy receipt were associated with first-line clinical trial enrollment odds. Patterns of associations for White patients were generally similar to the main results. In models where missing data were imputed (Online Resource 3), race-specific patterns of associations between predictors and clinical trial enrollment odds were similar to the main analysis.

## Discussion

In this study, we examined heterogeneity in the roles of patient, area-level, facility, tumor, and treatment characteristics with clinical trial enrollment odds among gynecologic cancer patients by race. Most predictors did not differ by race, and we noted few associations for the much smaller group of Black gynecologic cancer patients. Among the predictors for Black patients were a cervical cancer diagnosis, advanced stage, and more recent diagnosis. These findings are in line with the notion that first-line treatment trials are more prevalent for advanced stage tumors and reflect recent efforts to increase enrollment of marginalized groups in clinical trials. Among White gynecologic cancer patients, in addition to similar associations as Black patients for recency of diagnosis and tumor characteristics, we also observed that individual-level factors (*e.g.,* presence of comorbid condition) and characteristics of both area (*e.g.,* area-level education) and facility (*e.g.,* academic/research programs) were related to clinical trial enrollment odds. Although the dearth of associations among Black patients is influenced by the low number of Black patients enrolled in clinical trials, this race-based evaluation of clinical trial enrollment predictors provides a starting point in understanding predictors of clinical trial enrollment disparities.

Prior studies on the existence of racial disparities in clinical trial enrollment among gynecologic cancer patients consistently demonstrate lower enrollment among minoritized as compared with White patients [7–14]; however, there is very little work exploring the determinants of clinical trial enrollment [13, 15] and only one study formally examined effect modification by race [15]. In the latter study, Jorge et al. examined limited English language proficiency as a predictor of clinical trial enrollment among 2700 gynecologic cancer patients treated at an academic medical center and showed lower odds of enrollment associated with limited vs. moderate/high English proficiency. Although effect modification was not observed, likely due to low numbers of Asian (*n* = 215), Black (*n* = 95), and Hispanic (*n* = 174) patients, White and Black patients with limited English language proficiency had lower clinical trial enrollment compared with their counterparts who were moderately/highly proficient in English.

We previously examined predictors of clinical trial enrollment in a multiracial cohort of gynecologic cancer patients using the NCDB; however, we did not present race-specific associations [13]. Given the numerical predominance of White patients in that sample (79%), the unstratified estimates were mainly applicable to the majority group of White patients. Our current analysis of clinical trial predictors among White gynecologic cancer patients reflects the findings of the prior work, namely lower clinical trial enrollment odds were observed among older patients, those with comorbidities, areas with lower educational attainment, and treatment in the South and Midwest, while treatment at an academic/research program was associated with higher odds of enrollment. In the current analysis, our finding that more recently diagnosed gynecologic cancer patients (both Black and White) had higher clinical trial enrollment odds likely reflects increased attention to low cancer clinical trial enrollment broadly, and heightened awareness that marginalized groups are even less likely to be invited and participate in clinical trials [4, 25, 26]. The cancer site findings showing higher odds of enrollment in first-line treatment trials for ovarian and cervical as compared with uterine cancers potentially highlights an important gap in treatment development for uterine cancers.

At present, the scant body of literature on clinical trial enrollment predictors among gynecologic cancer patients has mainly focused on the role of individual-level determinants, with the present analysis including some area- and facility-level characteristics as predictors. Importantly, participation in clinical trials represents the successful navigation of a pathway characterized by barriers at multiple levels, the most important of which arguably operate upstream of individual patient choices. At the time of the cancer diagnosis, structural barriers – mainly the geographic availability of clinical trials – limit enrollment of approximately 50% of cancer patients [25]. When clinical trials are available either at the treating institution or within a manageable distance from the patient residence, restrictive eligibility criteria may further limit clinical trial enrollment of 20% of cancer patients [25]. Finally, in the presence of available trials and eligible patients, physicians must discuss and offer clinical trials to their patients before patients can decide whether to participate. At each point along this pathway, patients are excluded from participation and the extent to which these various barriers differentially impact participation of minoritized as compared with White gynecologic cancer patients is unknown. Importantly, a meta-analysis demonstrated that when Black and White cancer patients are offered participation in clinical trials, they accept at equal rates [27]. Thus, a characterization of the upstream factors that drive racially disparate clinical trial enrollment should be a major focus of future gynecologic cancer research.

Limitations of the current analysis include a lack of granular details regarding the clinical trials in which patients were enrolled (*e.g.,* specific treatment, trial phase, sponsor, therapeutic area), provider characteristics, or additional context of the lived patient environment. These unmeasured characteristics could differently influence first-line clinical trial enrollment odds among Black and White gynecologic cancer patients. The analysis is also limited to clinical trial enrollment during first line of treatment; thus, patients enrolled at the time of recurrence or progression, likely the most prevalent type of gynecologic trials currently available, are not denoted within this dataset, leading to under ascertainment of clinical trial enrollment. Low absolute numbers of other minority groups within the NCDB limited our ability to assess predictors of clinical trial enrollment among other racially minoritized groups (*i.e.,* American Indian/Alaska Native, Asian, Native Hawaiian/Pacific Islander, and Hispanic); however, future analyses in these populations are warranted. Despite these limitations, important strengths of this analysis include the careful attention to the role of missing data, with implementation of various models to assess the robustness of our results and an investigation of how patient characteristics relate to clinical trial enrollment for White and Black gynecologic cancer patients.

In summary, we observed few racial differences in associations of clinical trial enrollment predictors between Black and White gynecologic cancer patients. While the low number of Black patients enrolled in clinical trials likely underlies the imprecise associations for this subgroup, this analysis serves as an important baseline for future research aimed at identifying factors that impact clinical trial enrollment among minoritized groups.

## Supplementary Information

Below is the link to the electronic supplementary material.

Supplementary file1 (DOCX 48 KB)

## Data Availability

The data that support the findings of this study are available by submitting a proposal to the National Cancer Database at: https://www.facs.org/quality-programs/cancer/ncdb.

## References

[1] Unger JM, LeBlanc M, George S et al (2023) Population, clinical, and scientific impact of national cancer institute’s national clinical trials network treatment studies. J Clin Oncol 41:2020–2028 36480773 10.1200/JCO.22.01826 PMC10082246

[2] Adams DV, Long S, Fleury ME (2022) Association of remote technology use and other decentralization tools with patient likelihood to enroll in cancer clinical trials. JAMA Netw Open 5:e2220053 35788672 10.1001/jamanetworkopen.2022.20053 PMC9257574

[3] Unger JM, Shulman LN, Facktor MA, et al. (2024) National Estimates of the Participation of Patients With Cancer in Clinical Research Studies Based on Commission on Cancer Accreditation Data. J Clin Oncol. JCO2301030. 10.1200/JCO.23.01030 PMC11191051 38564681

[4] Unger JM, Cook E, Tai E et al (2016) The role of clinical trial participation in cancer research: barriers, evidence, and strategies. American Society of clinical oncology educational book. American Society of clinical oncology. Annual Meeting 35:185–198 10.14694/EDBK_156686 PMC5495113 27249699

[5] Grant SR, Lin TA, Miller AB, et al. (2020) Racial and Ethnic Disparities Among Participants in US-Based Phase 3 Randomized Cancer Clinical Trials. JNCI cancer spectrum. 4: pkaa060. 10.1093/jncics/pkaa060 PMC7667997 33225207

[6] Dunlop H, Fitzpatrick E, Kurti K et al (2022) Participation of patients from racial and ethnic minority groups in phase 1 early cancer drug development trials in the US, 2000–2018. JAMA Netw Open 5:e2239884 36326764 10.1001/jamanetworkopen.2022.39884 PMC9634497

[7] Scalici J, Finan MA, Black J et al (2015) Minority participation in Gynecologic Oncology Group (GOG) studies. Gynecol Oncol 138:441–444 26013697 10.1016/j.ygyno.2015.05.014 PMC4670048

[8] Mishkin G, Minasian LM, Kohn EC et al (2016) The generalizability of NCI-sponsored clinical trials accrual among women with gynecologic malignancies. Gynecol Oncol 143:611–616 27697287 10.1016/j.ygyno.2016.09.026

[9] Awad E, Paladugu R, Jones N et al (2020) Minority participation in phase 1 gynecologic oncology clinical trials: three decades of inequity. Gynecol Oncol 157:729–732 32173047 10.1016/j.ygyno.2020.03.002

[10] Grette KV, White AL, Awad EK et al (2021) Not immune to inequity: minority under-representation in immunotherapy trials for breast and gynecologic cancers. Int J Gynecol Cancer 31:1403–1407 34088749 10.1136/ijgc-2021-002557

[11] Wagar MK, Mojdehbakhsh RP, Godecker A et al (2022) Racial and ethnic enrollment disparities in clinical trials of poly(ADP-ribose) polymerase inhibitors for gynecologic cancers. Gynecol Oncol 165:49–52 35144798 10.1016/j.ygyno.2022.01.032

[12] Mattei LH, Robb L, Banning K et al (2022) Enrollment of individuals from racial and ethnic minority groups in gynecologic cancer precision oncology trials. Obstet Gynecol 140:654–661 36075065 10.1097/AOG.0000000000004917

[13] Khadraoui W, Meade CE, Backes FJ et al (2023) Racial and ethnic disparities in clinical trial enrollment among women with gynecologic cancer. JAMA Netw Open 6:e2346494 38060227 10.1001/jamanetworkopen.2023.46494 PMC10704282

[14] Richardson MT, Barry D, Steinberg JR et al (2024) Underrepresentation of racial and ethnic minority groups in gynecologic oncology: an analysis of over 250 trials. Gynecol Oncol 181:1–7 38096673 10.1016/j.ygyno.2023.12.001

[15] Jorge S, Masshoor S, Gray HJ et al (2023) Participation of patients with limited english proficiency in gynecologic oncology clinical trials. Journal of the National Comprehensive Cancer Network : JNCCN 21:27-32.e2 36634612 10.6004/jnccn.2022.7068

[16] Merkow RP, Rademaker AW, Bilimoria KY (2018) Practical guide to surgical data sets: national cancer database (NCDB). JAMA Surg 153:850–851 29617542 10.1001/jamasurg.2018.0492

[17] Bilimoria KY, Stewart AK, Winchester DP et al (2008) The National Cancer Data Base: a powerful initiative to improve cancer care in the United States. Ann Surg Oncol 15:683–690 18183467 10.1245/s10434-007-9747-3 PMC2234447

[18] von Elm E, Altman DG, Egger M et al (2007) The strengthening the reporting of observational studies in epidemiology (STROBE) statement: guidelines for reporting observational studies. Epidemiology 18:800–804 18049194 10.1097/EDE.0b013e3181577654

[19] Elshami M, Hue JJ, Hoehn RS et al (2022) A nationwide analysis of clinical trial participation for common hepato-pancreato-biliary malignancies demonstrates survival advantages for subsets of trial patients but disparities in and infrequency of enrollment. HPB (Oxford) 24:1280–1290 35063353 10.1016/j.hpb.2021.12.022

[20] White IR, Royston P, Wood AM (2011) Multiple imputation using chained equations: Issues and guidance for practice. Stat Med 30:377–399 21225900 10.1002/sim.4067

[21] van Buuren S, Groothuis-Oudshoorn K (2011) mice: multivariate imputation by chained equations in R. J Stat Softw 45:1–67

[22] Little RJA, Rubin DB. (2020) Statistical Analysis with Missing Data. 3rd ed: John Wiley & Sons, Inc.

[23] Molenberghs G, Kenward MG. (2007) Multiple Imputation. Missing Data in Clinical Studies. pp. 105–17.

[24] Robitzsch A, Grund S. (2024) miceadds: Some Additional Multiple Imputation Functions, Especially for ‘mice’. R package version 3.17–44.

[25] Unger JM, Vaidya R, Hershman DL et al (2019) Systematic review and meta-analysis of the magnitude of structural, clinical, and physician and patient barriers to cancer clinical trial participation. J Natl Cancer Inst 111:245–255 30856272 10.1093/jnci/djy221 PMC6410951

[26] Kim ES, Bruinooge SS, Roberts S et al (2017) Broadening eligibility criteria to make clinical trials more representative: American Society of clinical oncology and friends of cancer research joint research statement. J Clin Oncol 35:3737–3744 28968170 10.1200/JCO.2017.73.7916 PMC5692724

[27] Unger JM, Hershman DL, Till C et al (2021) “When offered to participate”: a systematic review and meta-analysis of patient agreement to participate in cancer clinical trials. J Natl Cancer Inst 113:244–257 33022716 10.1093/jnci/djaa155 PMC7936064

