# Supplementary material for: Determinants of first-line clinical trial enrollment among Black and White gynecologic cancer patients
Source: Cancer Causes Control. 2025 Feb 3;36(6):625–32. doi: 10.1007/s10552-025-01963-y (PMC12098198; doi:10.1007/s10552-025-01963-y)
Supplement: Supplementary file 1 — Supplementary file1 (DOCX 48 KB) [file 10552_2025_1963_MOESM1_ESM.docx]

**Online Resource: Determinants of clinical trial enrollment among Black and White gynecologic cancer patients, Cancer Causes & Control**

Autumn B. Carey, MPH^1^, Caitlin E. Meade, BS^1^, Britton Trabert, PhD^2,3^, Casey M. Cosgrove, MD^4^, Ashley S. Felix, PhD^1^

^1^Division of Epidemiology, College of Public Health, The Ohio State University, Columbus, OH.

^2^Department of Obstetrics and Gynecology, Spencer Fox Eccles School of Medicine, University of Utah, Salt Lake City, UT.

^3^Huntsman Cancer Institute, University of Utah, Salt Lake City, UT.

^4^Department of Obstetrics and Gynecology, Division of Gynecologic Oncology, The Ohio State University Wexner Medical Center, Arthur G James Cancer Center, Columbus, OH.

**Corresponding author:**

Ashley S. Felix, PhD

Division of Epidemiology

College of Public Health

Ohio State University

1841 Neil Ave, Cunz Hall 304

Columbus, OH, 43210

[Ashley.Felix @osumc.edu](mailto:Ashley.Felix%20@osumc.edu)

(614) 688-1477

**Online Resource 1**

**Supplemental Methods**

Additional cancer site-specific exclusions were as follows: endometrial cancer (unknown if surgery performed, no surgery performed); cervical cancer (surgery types of local tumor destruction: photodynamic therapy or local tumor destruction: thermal ablation); peritoneal cancer (surgery types of local tumor destruction, NOS, local tumor destruction: laser, any combination of local tumor excision, polypectomy, or excisional biopsy with cryosurgery, any combination of local tumor excision, polypectomy, or excisional biopsy with laser ablation, any combination of local tumor excision, polypectomy, or excisional biopsy with electrocautery, or local tumor excision: polypectomy); and fallopian tube (surgery types of any combination of local tumor excision, polypectomy, or excisional biopsy with electrocautery, any combination of local tumor excision, polypectomy, excisional biopsy with laser ablation, any combination of local tumor excision, polypectomy, excisional biopsy with cryosurgery, local tumor excision: polypectomy, Local tumor destruction, NOS, or local tumor excision: laser excision).

| **Online Resource 2.** Multivariable-adjusted odds ratios (ORs) and 95% confidence intervals (CIs) for associations between epidemiologic, facility, and tumor, and first-line treatment characteristics and clinical trial enrollment by race (missing data excluded) | | | | | | | | | |
| --- | --- | --- | --- | --- | --- | --- | --- | --- | --- |
|  | **Black (n=62,209)** | | | | **White (n=459,591)** | | | | |
|  | **Not Enrolled (n=62,170)** | **Enrolled (n=39)** | **OR (95% CI)** | **p** | **Not Enrolled (n=459,040)** | **Enrolled (n=551)** | **OR (95% CI)** | **p** | **P_int_^a^** |
| **Age, mean (SD)** | 62.01 (10.87) | 59.26 (10.49) | 1.00 (0.83, 1.21) | 1.00 | 63.10 (11.17) | 61.15 (9.52) | 0.89 (0.84, 0.94) | <0.0001 | 0.96 |
| **Charlson-Deyo score** |  |  |  | 0.63 |  |  |  | 0.03 | 0.22 |
| 0 | 43,898 (70.6) | 27 (69.2) | 1.00 |  | 354,096 (77.1) | 463 (84.0) | 1.00 |  |  |
| 1 | 12,978 (20.9) | 8 (20.5) | 1.28 (0.57, 2.85) |  | 78,860 (17.2) | 75 (13.6) | 0.95 (0.75, 1.22) |  |  |
| ≥2 | 5,294 (8.5) | 4 (10.3) | 1.59 (0.54, 4.67) |  | 26,084 (5.7) | 13 (2.4) | 0.47 (0.27, 0.82) |  |  |
| **Insurance Status** |  |  |  | 0.12 |  |  |  | 0.38 | 0.71 |
| No insurance | 3,908 (6.3) | 3 (7.7) | 0.78 (0.23, 2.67) |  | 12,967 (2.8) | 15 (2.7) | 1.02 (0.60, 1.73) |  |  |
| Private insurance | 23,936 (38.5) | 21 (53.9) | 1.00 |  | 223,303 (48.7) | 306 (55.5) | 1.00 |  |  |
| Medicaid | 9,065 (14.6) | 6 (15.4) | 0.47 (0.18, 1.20) |  | 25,637 (5.6) | 26 (4.7) | 0.67 (0.45, 1.01) |  |  |
| Medicare | 24,643 (39.6) | 8 (20.5) | 0.33 (0.13, 0.85) |  | 192,682 (42.0) | 197 (35.8) | 0.94 (0.74, 1.20) |  |  |
| Other government | 618 (1.0) | 1 (2.6) | 1.70 (0.22, 13.26) |  | 4,451 (1.0) | 7 (1.3) | 1.23 (0.58, 2.63) |  |  |
| **Distance from facility, miles** |  |  |  | 0.007 |  |  |  | 0.002 | 0.05 |
| <7.6 miles | 29,648 (47.7) | 20 (51.3) | 1.00 |  | 147,478 (32.1) | 128 (23.2) | 1.00 |  |  |
| 7.6-23.4 miles | 19,494 (31.4) | 7 (18.0) | 0.62 (0.25, 1.53) |  | 158,710 (34.6) | 211 (38.3) | 1.33 (1.07, 1.67) |  |  |
| >23.4 miles | 13,028 (21.0) | 12 (30.8) | 3.33 (1.28, 8.64) |  | 152,852 (33.3) | 212 (38.5) | 1.54 (1.20, 1.96) |  |  |
| **Area-level income** |  |  |  | 0.01 |  |  |  | 0.20 | 0.10 |
| Quartile 1: $46,277 | 25,850 (41.6) | 17 (43.6) | 1.00 |  | 61,050 (13.3) | 53 (9.6) | 1.00 |  |  |
| Quartile 2: $46,277-$57,856 | 13,901 (22.4) | 14 (35.9) | 1.14 (0.51, 2.55) |  | 101,714 (22.2) | 108 (19.6) | 1.08 (0.76, 1.52) |  |  |
| Quartile 3: $57,857-$74,062 | 10,779 (17.3) | 5 (12.8) | 0.39 (0.12, 1.20) |  | 115,154 (25.1) | 127 (23.1) | 0.88 (0.62, 1.27) |  |  |
| Quartile 4: >= $74,063 | 11,640 (18.7) | 3 (7.7) | 0.14 (0.03, 0.65) |  | 181,122 (39.5) | 263 (47.7) | 0.78 (0.53, 1.16) |  |  |
| **Area-level education (% without high school diploma)** |  |  |  | 0.04 |  |  |  | 0.006 | 0.57 |
| Quartile 1: < 5% | 4,253 (6.8) | 2 (5.1) | 1.00 |  | 112,052 (24.4) | 181 (32.9) | 1.00 |  |  |
| Quartile 2: 5% - 9% | 10,865 (17.5) | 11 (28.2) | 1.22 (0.25, 6.01) |  | 146,857 (32.0) | 183 (33.2) | 0.82 (0.66, 1.03) |  |  |
| Quartile 3: 9.1%-15.2% | 22,246 (35.8) | 13 (33.3) | 0.42 (0.08, 2.18) |  | 127,961 (27.9) | 117 (21.2) | 0.62 (0.47, 0.81) |  |  |
| Quartile 4: ≥15.3% | 24,806 (39.9) | 13 (33.3) | 0.31 (0.06, 1.75) |  | 72,170 (15.7) | 70 (12.7) | 0.66 (0.47, 0.93) |  |  |
| **Metro status** |  |  |  | 0.04 |  |  |  | 0.0002 | 0.12 |
| Large metro county (population > 1 million) | 41,853 (67.3) | 32 (82.1) | 1.00 |  | 228,269 (49.7) | 332 (60.3) | 1.00 |  |  |
| Medium/small metro county (population < 1 million) | 14,275 (23.0) | 2 (5.1) | 0.12 (0.03, 0.52) |  | 151,268 (33.0) | 152 (27.6) | 0.69 (0.56, 0.86) |  |  |
| Urban | 5,358 (8.6) | 4 (10.3) | 0.53 (0.14, 1.95) |  | 70,971 (15.5) | 57 (10.3) | 0.51 (0.37, 0.71) |  |  |
| Rural | 684 (1.1) | 1 (2.6) | 0.96 (0.11, 8.58) |  | 8,532 (1.9) | 10 (1.8) | 0.80 (0.41, 1.56) |  |  |
| **Facility location** |  |  |  | 0.51 |  |  |  | 0.04 | 0.34 |
| Northeast | 12,620 (20.3) | 10 (25.6) | 1.00 |  | 102,848 (22.4) | 169 (30.7) | 1.00 |  |  |
| South | 34,491 (55.5) | 16 (41.0) | 0.51 (0.20, 1.27) |  | 154,484 (33.7) | 156 (28.3) | 0.71 (0.56, 0.90) |  |  |
| Midwest | 11,124 (17.9) | 12 (30.8) | 1.01 (0.41, 2.49) |  | 124,027 (27.0) | 134 (24.3) | 0.77 (0.60, 0.97) |  |  |
| Mountain | 489 (0.8) | 0 (0.0) | - |  | 20,173 (4.4) | 26 (4.7) | 0.98 (0.64, 1.50) |  |  |
| Pacific | 3,446 (5.5) | 1 (2.6) | 0.59 (0.07, 4.80) |  | 57,508 (12.5) | 66 (12.0) | 0.84 (0.62, 1.12) |  |  |
| **Facility type** |  |  |  | 0.22 |  |  |  | <0.0001 | 0.27 |
| Community Cancer Program | 1,713 (2.8) | 1 (2.6) | 1.00 |  | 18,322 (4.0) | 10 (1.8) | 1.00 |  |  |
| Comprehensive Community Cancer Program | 17,252 (27.8) | 3 (7.7) | 0.29 (0.03, 2.87) |  | 173,562 (37.8) | 111 (20.1) | 1.01 (0.53, 1.95) |  |  |
| Academic/Research Program | 30,741 (49.5) | 27 (69.2) | 1.04 (0.14, 7.80) |  | 171,205 (37.3) | 355 (64.4) | 2.58 (1.36, 4.89) |  |  |
| Integrated Network Cancer Program | 12,464 (20.1) | 8 (20.5) | 0.77 (0.09, 6.36) |  | 95,951 (20.9) | 75 (13.6) | 1.11 (0.57, 2.16) |  |  |
| **Diagnosis year** |  |  |  | 0.14 |  |  |  | <0.0001 | 0.07 |
| 2004-2007 | 9,772 (15.7) | 1 (2.6) | 1.00 |  | 84,167 (18.3) | 33 (6.0) | 1.00 |  |  |
| 2008-2011 | 12,871 (20.7) | 10 (25.6) | 7.41 (0.95, 58.09) |  | 99,955 (21.8) | 81 (14.7) | 2.14 (1.43, 3.21) |  |  |
| 2012-2015 | 16,500 (26.5) | 9 (23.1) | 5.15 (0.65, 40.89) |  | 119,656 (26.1) | 88 (16.0) | 1.99 (1.33, 2.98) |  |  |
| 2015-2020 | 23,027 (37.0) | 19 (48.7) | 8.62 (1.14, 65.01) |  | 155,262 (33.8) | 349 (63.3) | 6.75 (4.71, 9.66) |  |  |
| **Cancer site** |  |  |  | 0.03 |  |  |  | <0.0001 | 0.01 |
| Uterine | 34,762 (55.9) | 11 (28.2) | 1.00 |  | 268,749 (58.6) | 70 (12.7) | 1.00 |  |  |
| Ovarian | 14,144 (22.8) | 16 (41.0) | 1.37 (0.59, 3.21) |  | 138,755 (30.2) | 443 (80.4) | 3.84 (2.77, 5.32) |  |  |
| Cervical | 13,264 (21.3) | 12 (30.8) | 4.79 (1.53, 14.98) |  | 51,536 (11.2) | 38 (6.9) | 2.68 (1.69, 4.25) |  |  |
| **Tumor stage** |  |  |  | 0.009 |  |  |  | <0.0001 | 0.92 |
| I | 29,172 (46.9) | 3 (7.7) | 1.00 |  | 254,148 (55.4) | 33 (6.0) | 1.00 |  |  |
| II | 6,366 (10.2) | 2 (5.1) | 2.05 (0.31, 13.47) |  | 37,340 (8.1) | 35 (6.4) | 4.25 (2.56, 7.08) |  |  |
| III | 15,334 (24.7) | 20 (51.3) | 8.04 (2.08, 31.15) |  | 107,723 (23.5) | 335 (60.8) | 10.37 (6.87, 15.66) |  |  |
| IV | 11,298 (18.2) | 14 (35.9) | 7.46 (1.84, 30.30) |  | 59,829 (13.0) | 148 (26.9) | 8.89 (5.78, 13.67) |  |  |
| **Surgery** |  |  |  | 0.14 |  |  |  | <0.0001 | 0.18 |
| No surgery | 12,450 (20.0) | 10 (25.6) | 1.00 |  | 52,460 (11.4) | 47 (8.5) | 1.00 |  |  |
| Any surgery | 49,720 (80.0) | 29 (74.4) | 2.09 (0.79, 5.52) |  | 406,580 (88.6) | 504 (91.5) | 2.62 (1.85, 3.72) |  |  |
| **Chemotherapy** |  |  |  | 0.02 |  |  |  | 0.0001 | 0.67 |
| No | 31,762 (51.1) | 4 (10.3) | 1.00 |  | 269,082 (58.6) | 56 (10.2) | 1.00 |  |  |
| Yes | 30,408 (48.9) | 35 (89.7) | 4.08 (1.26, 13.22) |  | 189,958 (41.4) | 495 (89.8) | 1.89 (1.37, 2.61) |  |  |
| **Radiation** |  |  |  | 0.09 |  |  |  | 0.16 | 0.19 |
| No | 41,298 (66.4) | 28 (71.8) | 1.00 |  | 343,320 (74.8) | 490 (88.9) | 1.00 |  |  |
| Yes | 20,872 (33.6) | 11 (28.2) | 0.43 (0.16, 1.15) |  | 115,720 (25.2) | 61 (11.1) | 0.75 (0.51, 1.12) |  |  |
| a interaction between race and the assessed variable | | | | | | | | | |

| **Online Resource 3.** Multivariable-adjusted odds ratios (ORs) and 95% confidence intervals (CIs) for associations between epidemiologic, facility, and tumor, and first-line treatment characteristics and clinical trial enrollment by race (using multiple imputation by chained equations to impute missing data) | | | | | | | | | |
| --- | --- | --- | --- | --- | --- | --- | --- | --- | --- |
|  | **Black (n=86,058)** | | | | **White (n=616,964)** | | | | |
|  | **Not Enrolled (n=86,009)** | **Enrolled (n=49)** | **OR (95% CI)** | **p** | **Not Enrolled (n=616,254)** | **Enrolled (n=710)** | **OR (95% CI)** | **p** | **P_int_^a^** |
| **Age, mean (SD)** | 59.44 (13.39) | 56.98 (12.50) | 1.04 (0.91, 1.19) | 0.20 | 61.11 (13.09) | 59.86 (11.06) | 0.93 (0.90, 0.97) | 0.01 | 0.73 |
| **Charlson-Deyo score** |  |  |  | 0.59 |  |  |  | <0.0001 | 0.18 |
| 0 | 62,077 (72.2) | 33 (67.4) | 1.00 |  | 480,188 (77.9) | 599 (84.4) | 1.00 |  |  |
| 1 | 17,050 (19.8) | 12 (24.5) | 1.68 (0.86, 3.28) |  | 102,531 (16.6) | 94 (13.2) | 0.95 (0.77, 1.18) |  |  |
| ≥2 | 6,882 (8.0) | 4 (8.2) | 1.39 (0.48, 4.01) |  | 33,535 (5.4) | 17 (2.4) | 0.49 (0.30, 0.80) |  |  |
| **Insurance Status** |  |  |  | 0.06 |  |  |  | 0.05 | 0.27 |
| No insurance | 5,400 (6.3) | 4 (8.2) | 0.90 (0.31, 2.66) |  | 18,239 (3.0) | 16 (2.3) | 0.84 (0.51, 1.40) |  |  |
| Private insurance | 33,459 (38.9) | 26 (53.1) | 1.00 |  | 303,199 (49.2) | 404 (56.9) | 1.00 |  |  |
| Medicaid | 13,525 (15.7) | 10 (20.4) | 0.64 (0.30, 1.37) |  | 39,552 (6.4) | 38 (5.4) | 0.70 (0.49, 0.99) |  |  |
| Medicare | 30,886 (35.9) | 8 (16.3) | 0.26 (0.11, 0.63) |  | 239,950 (38.9) | 240 (33.8) | 0.81 (0.65, 1.01) |  |  |
| Other government | 903 (1.1) | 1 (2.0) | 1.22 (0.16, 9.20) |  | 6,289 (1.0) | 7 (1.0) | 0.88 (0.42, 1.85) |  |  |
| **Distance from facility, miles** |  |  |  | 0.02 |  |  |  | <0.0001 | 0.05 |
| <7.6 miles | 35,490 (41.3) | 23 (46.9) | 1.00 |  | 168,661 (27.4) | 139 (19.6) | 1.00 |  |  |
| 7.6-23.4 miles | 23,869 (27.8) | 8 (16.3) | 0.57 (0.24, 1.32) |  | 184,882 (30.0) | 235 (33.1) | 1.37 (1.11, 1.68) |  |  |
| >23.4 miles | 16,723 (19.4) | 13 (26.5) | 2.62 (1.17, 5.86) |  | 195,042 (31.7) | 261 (36.8) | 1.60 (1.28, 2.01) |  |  |
| **Area-level income** |  |  |  | 0.32 |  |  |  | <0.0001 | 0.14 |
| Quartile 1: <$46,277 | 31,394 (36.5) | 20 (40.8) | 1.00 |  | 73,582 (11.9) | 59 (8.3) | 1.00 |  |  |
| Quartile 2: $46,277-$57,856 | 16,738 (19.5) | 14 (28.6) | 0.87 (0.40, 1.90) |  | 120,750 (19.6) | 121 (17.0) | 0.95 (0.41, 2.21) |  |  |
| Quartile 3: $57,857-$74,062 | 13,052 (15.2) | 5 (10.2) | 0.31 (0.1, 0.93) |  | 135,939 (22.1) | 138 (19.4) | 1.00 (0.36, 2.77) |  |  |
| Quartile 4: ≥ $74,063 | 13,853 (16.1) | 5 (10.2) | 0.25 (0.07, 0.90) |  | 211,110 (34.3) | 306 (43.1) | 0.85 (0.28, 2.60) |  |  |
| **Area-level education (% without high school diploma)** |  |  |  | 0.04 |  |  |  | 0.12 | 0.19 |
| Quartile 1: < 5% | 5,096 (5.9) | 2 (4.1) | 0.49 (0.09, 2.68) |  | 131,414 (21.3) | 209 (29.4) | 0.64 (0.36, 1.14) |  |  |
| Quartile 2: 5% - 9% | 13,108 (15.2) | 13 (26.5) | 0.76 (0.14, 4.04) |  | 172,338 (28.0) | 209 (29.4) | 0.78 (0.44, 1.37) |  |  |
| Quartile 3: 9.1%-15.2% | 26,787 (31.1) | 15 (30.6) | 1.84 (0.38, 8.83) |  | 151,879 (24.7) | 135 (19.0) | 0.91 (0.67, 1.25) |  |  |
| Quartile 4: ≥15.3% | 30,203 (35.1) | 14 (28.6) | 1.00 |  | 87,459 (14.2) | 74 (10.4) | 1.00 |  |  |
| **Metro status** |  |  |  | 0.04 |  |  |  | <0.0001 | 0.68 |
| Large metro county (population > 1 million) | 56,975 (66.2) | 36 (73.5) | 1.00 |  | 292,952 (47.5) | 398 (56.1) | 1.00 |  |  |
| Medium/small metro county (population < 1 million) | 19,093 (22.2) | 7 (14.3) | 0.40 (0.16, 1.00) |  | 193,354 (31.4) | 197 (27.8) | 0.72 (0.56, 0.93) |  |  |
| Urban | 7,001 (8.1) | 4 (8.2) | 0.48 (0.14, 1.66) |  | 92,784 (15.1) | 72 (10.1) | 0.50 (0.33, 0.76) |  |  |
| Rural | 896 (1.0) | 1 (2.0) | 0.96 (0.11, 8.14) |  | 11,323 (1.8) | 13 (1.8) | 0.95 (0.53, 1.71) |  |  |
| **Facility location** |  |  |  | 0.26 |  |  |  | <0.0001 | 0.39 |
| Northeast | 15,347 (17.8) | 11 (22.5) | 1.00 |  | 127,986 (20.8) | 211 (29.7) | 1.00 |  |  |
| South | 44,393 (51.6) | 20 (40.8) | 0.56 (0.23, 1.35) |  | 193,695 (31.4) | 185 (26.1) | 0.66 (0.52, 0.84) |  |  |
| Midwest | 14,028 (16.3) | 13 (26.5) | 0.98 (0.41, 2.37) |  | 160,870 (26.1) | 169 (23.8) | 0.72 (0.57, 0.91) |  |  |
| Mountain | 639 (0.7) | 0 (0.0) | NE |  | 27,759 (4.5) | 36 (5.1) | 0.96 (0.66, 1.39) |  |  |
| Pacific | 3,909 (4.5) | 1 (2.0) | 0.48 (0.06, 3.81) |  | 65,926 (10.7) | 73 (10.3) | 0.88 (0.62, 1.25) |  |  |
| **Facility type** |  |  |  | 0.17 |  |  |  | <0.0001 | 0.61 |
| Community Cancer Program | 2,097 (2.4) | 1 (2.0) | 1.00 |  | 21,740 (3.5) | 12 (1.7) | 1.00 |  |  |
| Comprehensive Community Cancer Program | 21,768 (25.3) | 5 (10.2) | 0.39 (0.05, 3.28) |  | 211,910 (34.4) | 144 (20.3) | 1.11 (0.61, 1.99) |  |  |
| Academic/Research Program | 38,164 (44.4) | 31 (63.3) | 0.96 (0.13, 7.09) |  | 220,842 (35.8) | 424 (59.7) | 2.48 (1.38, 4.47) |  |  |
| Integrated Network Cancer Program | 16,287 (18.9) | 8 (16.3) | 0.62 (0.07, 5.14) |  | 121,744 (19.8) | 94 (13.2) | 1.11 (0.60, 2.03) |  |  |
| **Diagnosis year** |  |  |  | 0.005 |  |  |  | <0.0001 | 0.12 |
| 2004-2007 | 13,073 (15.2) | 1 (2.0) | 1.00 |  | 109,808 (17.8) | 44 (6.2) | 1.00 |  |  |
| 2008-2011 | 17,791 (20.7) | 11 (22.5) | 8.08 (1.03, 63.31) |  | 133,798 (21.7) | 103 (14.5) | 1.95 (1.37, 2.78) |  |  |
| 2012-2015 | 23,009 (26.8) | 11 (22.5) | 6.30 (0.80, 49.30) |  | 162,534 (26.4) | 117 (16.5) | 1.86 (1.31, 2.65) |  |  |
| 2015-2020 | 32,136 (37.4) | 26 (53.1) | 12.18 (1.65, 89.95) |  | 210,114 (34.1) | 446 (62.8) | 6.05 (4.42, 8.28) |  |  |
| **Cancer site** |  |  |  | <0.0001 |  |  |  | <0.0001 | 0.005 |
| Uterine | 45,526 (52.9) | 12 (24.5) | 1.00 |  | 345,223 (56.0) | 94 (13.2) | 1.00 |  |  |
| Ovarian | 19,662 (22.9) | 21 (42.9) | 1.79 (0.80, 3.99) |  | 184,190 (29.9) | 564 (79.4) | 3.25 (2.47, 4.28) |  |  |
| Cervical | 20,821 (24.2) | 16 (32.7) | 4.76 (1.72, 13.19) |  | 86,841 (14.1) | 52 (7.3) | 2.16 (1.46, 3.20) |  |  |
| **Tumor stage** |  |  |  | <0.0001 |  |  |  | <0.0001 | 0.86 |
| I | 40,923 (47.6) | 4 (8.2) | 1.00 |  | 343,153 (55.7) | 43 (6.1) | 1.00 |  |  |
| II | 9,012 (10.5) | 3 (6.1) | 2.41 (0.49, 11.79) |  | 50,754 (8.2) | 49 (6.9) | 4.81 (3.12, 7.40) |  |  |
| III | 21,043 (24.5) | 26 (53.1) | 8.50 (2.57, 28.10) |  | 144,212 (23.4) | 405 (57.0) | 9.97 (6.87, 14.47) |  |  |
| IV | 15,031 (17.5) | 16 (32.7) | 7.24 (2.11, 24.90) |  | 78,135 (12.7) | 213 (30.0) | 10.38 (7.15, 15.07) |  |  |
| **Surgery** |  |  |  | 0.04 |  |  |  | <0.0001 | 0.10 |
| No surgery | 17,544 (20.4) | 13 (26.5) | 1.00 |  | 71,406 (11.6) | 61 (8.6) | 1.00 |  |  |
| Any surgery | 68,465 (79.6) | 36 (73.5) | 2.10 (0.90 4.87) |  | 544,848 (88.4) | 649 (91.4) | 2.56 (1.91, 3.43) |  |  |
| **Chemotherapy** |  |  |  | 0.03 |  |  |  | <0.0001 | 0.94 |
| No | 42,889 (49.9) | 6 (12.2) | 1.00 |  | 352,448 (57.2) | 68 (9.6) | 1.00 |  |  |
| Yes | 41,267 (48.0) | 43 (87.8) | 3.03 (1.14, 8.08) |  | 253,025 (41.1) | 642 (90.4) | 2.20 (1.64, 2.96) |  |  |
| **Radiation** |  |  |  | 0.15 |  |  |  | 0.02 | 0.05 |
| No | 55,286 (64.3) | 34 (69.4) | 1.00 |  | 446,174 (72.4) | 616 (86.8) | 1.00 |  |  |
| Yes | 27,886 (32.4) | 15 (30.6) | 0.52 (0.22, 1.26) |  | 150,928 (24.5) | 76 (10.7) | 0.66 (0.47, 0.92) |  |  |
| a interaction between race and the assessed variable | | | | | | | | | |
| NE: Not estimable | | |  |  |  |  |  |  |  |
